# Co-culture with *Lactobacillus plantarum* SC-1 facilitates ergosterol synthesis in *Monascus purpureus* through MpSet1-affected H3K4ac establishment

**DOI:** 10.3389/fmicb.2025.1603805

**Published:** 2025-06-23

**Authors:** Zhongling Wu, Zhenmin Liu

**Affiliations:** State Key Laboratory of Dairy Biotechnology Shanghai Engineering Research Center of Dairy Biotechnology, Dairy Research Institute, Bright Dairy & Food Co., Ltd., Shanghai, China

**Keywords:** co-culture, *Monascus purpureus*, ergosterol, *Lactobacillus plantarum*, H3K4ac

## Abstract

**Introduction:**

In fungi, ergosterol synthesis is regulated by multiple transcription factors such as Upc2, but the regulation of ergosterol synthesis in *Monascus* remains unclear.

**Methods:**

We performed a co-culture system of Monascus purpureus (*M. purpureus*) with Lactobacillus plantarum SC-1 strain isolated from sauerkraut to detect ergosterol content in *M. purpureus*. RNA high-throughput sequencing and Western blotting were used to analyze gene expression and histone modification levels in *M. purpureus*.

**Results:**

We found that co-culturing M. purpureus with SC-1 strain, resulted in a 45.1% increase in ergosterol content in *M. purpureus*. In addition, the transcription of ergosterol synthesis-related genes (ERGs) in *M. purpureus* was activated upon co-culture with SC-1 strain, accompanied by an increase in H3Kac levels. Interestingly, we further found that histone methyltransferase MpSet1 negatively regulated ergosterol synthesis in *M. purpureus*. Deletion of *MpSET1* led to the activation of ERGs transcription and the increase of H3K4ac levels. Moreover, 45% of the upregulated differentially expressed genes (up_DEGs) in the wild-type (WT) co-cultured with SC-1 strain overlapped with the up_DEGs in the *Δset1* strain, indicating that MpSet1 plays an important role in facilitating ergosterol synthesis in WT co-cultured with SC-1 strain.

**Conclusion:**

The study reveal that microbial co-cultivation can be used to facilitate ergosterol synthesis, and Set1 plays an important role in the ergosterol synthesis in fungi through affecting H3K4ac establishment.

## 1 Introduction

Ergosterol, a steroid unique to fungi, is an important component of fungal cell membranes, playing an essential role in maintaining cell membrane integrity, fluidity, enzyme binding activity, and intracellular transport (Jorda and Puig, 2020; Wollam and Antebi, 2011). In agriculture, many antifungal drugs are designed to target key reaction enzymes in the ergosterol synthesis pathway, thereby inhibiting the ergosterol synthesis in pathogenic fungi, disrupting fungal biofilm formation, and ultimately suppressing fungal growth (Zobi and Algul, 2025; Tanwar et al., 2024). In the field of medicine and health, ergosterol serves as a synthetic precursor of vitamin D2, is a main raw material for the production of synthetic steroid hormone and novel anticancer drugs, and it exhibits antibacterial, anti-inflammatory and anti-tumor effects (Martinez-Burgos et al., 2024; Wang et al., 2024). Consequently, ergosterol is recognized as a bioactive compound of great economic value. It is crucial to understand the synthesis regulation of ergosterol and increase ergosterol production.

In mammals, sterol synthesis is regulated by the sterol regulatory element-binding protein (Srebp) and its associated cleavage-activating protein (Scap) (Shao and Espenshade, 2014; Shimano, 2001). Srebp is a transcription factor associated with the endoplasmic reticulum, characterized by a classic helix–loop–helix domain and two transmembrane domains (Liu Z. et al., 2019; Yokoyama et al., 1993). Scap contains eight transmembrane segments at the N-terminus and a WD repeat domain at the C-terminus (Cheng et al., 2022). Under sterol-sufficient conditions, Scap binds directly to cholesterol, promoting its interaction with the insulin-induced gene protein (Insig), thereby keeping the SCAP-SREBP complex in the endoplasmic reticulum (Shao and Espenshade, 2014; Esquejo et al., 2021; Wang et al., 2023). However, in sterol-depleted cells, Scap dissociates from Insig, enabling it to escort Srebp from the ER to the Golgi. SREBP undergoes sequential proteolytic cleavage by Site 1 and Site 2 proteases, releasing its N-terminal transcription factor domain from the membrane. The liberated Srebp dimer then translocates into the nucleus via importin *β* and binds to the promoters of cholesterol metabolism-related target genes (Chandrasekaran and Weiskirchen, 2024; McPherson and Gauthier, 2004; Shimano and Sato, 2017).

In fungi, ergosterol synthesis is regulated by multiple regulatory factors (Hu et al., 2017; Liu Z. et al., 2019). For example, in *Saccharomyces cerevisiae*, the transcription factor Upc2 regulates ergosterol synthesis. Similar to Srebp, Upc2 is a membrane-bound transcription factor that becomes activated when ergosterol synthesis is blocked (Jorda and Puig, 2020; Vik and Rine, 2001). However, unlike Srebp, the C-terminal domain of Upc2 is a ligand binding domain that can bind to ergosterol (Yang et al., 2015). When Upc2 binds to ergosterol, it is localized in the cytoplasm. When the ergosterol content decreases, Upc2 separated from ergosterol enters the nucleus and globally activates the transcriptions of ergosterol synthesis-related genes (ERGs), promoting ergosterol synthesis and allowing the intracellular ergosterol content to reach a steady state (Marie et al., 2008). Upc2 mutant ligand binding domain increase yeast resistance to sterol synthesis inhibitors, indicating that Upc2 can be used as a target for increasing ergosterol synthesis production (Yang et al., 2015). In the pathogenic fungus *Fusarium graminearum*, Upc2 family homologous proteins are not involved in the regulation of ergosterol synthesis, but a pioneer transcription factor FgSr recruits the chromatin remodeling complex SWI/SNF to regulate ERGs transcriptions and mediate ergosterol synthesis, however, FgSR homologous protein is only conserved in the fungi of Sordella and Hammer Glossata classes (Liu Z. et al., 2019). In *Cryptococcus neoformans*, *Schizosaccharomyces pombe* and *Aspergillus fumigatus*, the regulatory mechanism mediated by Srebp orthologs is similar to that in mammals (Madhani et al., 2011; Chang et al., 2007; Gómez et al., 2021).

*Monascus purpureus* (*M. purpureus*), an important food microorganism, has been used for millennia in Southeast Asia (Wu et al., 2024). *M. purpureus* can produce beneficial secondary metabolites such as *Monascus* pigments (MPs), Monacolin K and ergosterol during fermentation, which exhibit not only good colorability but also functional activities, including anti-cancer and anti-inflammatory (Wu et al., 2024; Patakova, 2013; Vendruscolo et al., 2015; Agboyibor et al., 2018; Chen et al., 2017; Feng et al., 2016), these indicates that *Monascus* metabolites have potential for application in various sectors. In *M. purpureus*, double deletion of *EGR4* reduced ergosterol concentration while enhancing extracellular MP production (Liu J. et al., 2019). However, the synthesis regulation mechanism of ergosterol in *M. purpureus* is still unclear. In addition, Studies have shown that co-culturing *M. purpureus* with lactic acid bacteria can enhance the production of MPs and other functional compounds (Wu et al., 2023), however, whether the ergosterol synthesis can be facilitated by such co-culture is still unclear.

In this study, we identified a strain of *Lactobacillus plantarum* (*L. plantarum*) SC-1 co-cultured with *M. purpureus* that can facilitate the ergosterol synthesis in *M. purpureus*. The transcriptions of ERGs were activated, the histone H3kac level was increased, and the content of ergosterol was increased in *M. purpureus* co-cultured with SC-1 strain. In addition, MpSet1 was found to play a role in this regulatory process. Loss of *MpSET1* led to the activation of ERGs transcriptions and increased H3K4ac levels, thereby facilitating ergosterol synthesis.

## 2 Materials and methods

### 2.1 Strains and growth conditions

The wild-type (WT) strain (NRRL1596) of *M. purpureus*, the *SET1* gene knockout mutant (Δ*set1*) of *M. purpureus*, and *L. plantarum* SC-1, which was isolated from sauerkraut, are preserved at our research institute. The SC-1 strain was cultured on De Man-Rogosa-Sharpe (MRS) agar medium at 30°C for 24 h. For colony morphology analysis, the fungal strains were cultured on potato dextrose agar (PDA) medium at 30°C for 7 days to assess the growth.

To investigate the role of co-culturing SC-1 with *M. purpureus*, SC-1 strain was cultured in MRS liquid medium until reaching a concentration of 10^8^ colony-forming units per milliliter (cfu mL^−1^), which was diluted to 10^3^ cfu mL^−1^, and 1 mL of this diluted suspension was centrifuged. The resulting pellet was then resuspended and applied to PDA medium, co-culturing with *M. purpureus* at 30°C for 7 days.

To evaluate the role of SC-1 on ergosterol synthesis in *M. purpureus*, both the WT and WT co-cultured with SC-1 strains were grown on PDA or PDA supplemented 5 μg mL^−1^ triadimefon, an inhibitor of ergosterol synthesis, for 7 days. The relative inhibition to triadimefon rate was calculated using the following formula:


$$\text{Relative inhibition to triadimefon rate}/\%=\frac{A2-A1}{A1}\times 100.$$

In the formula, *A*1 represents the colony diameter (cm) of the strain grown on PDA without triadimefon. *A*2 represents the colony diameter of the strain grown on PDA containing triadimefon.

### 2.2 Detection of ergosterol

To detect the ergosterol content, 24 h PDB culture broths of SC-1 strain were mixed with equal volumes of PDB broth containing 24 h cultures of *M. purpureus* and co-cultured for 24 h, 1 g of mycelial tissue was ground and added to 2 mL of 3 M KOH in ethanol and incubated at 70°C for 1 h. The extraction mixture was centrifuged at 4,000 rpm for 15 min and the supernatant was diluted with 1 mL of distilled water. Ergosterol was extracted from the supernatant by using 2.5 mL of hexane twice in succession. The cyclohexane layer was concentrated and dried under vacuum at 40°C and then dissolved in 200 μL of methanol. The solution was filtered through a 0.45 μm microporous membrane and analyzed for ergosterol content by using a Waters 2696 HPLC system.

Liquid chromatography conditions: isocratic elution for 30 min; column temperature: 30°C; flow rate: 1.0 mL/min; injection volume: 5 μL; chromatographic column: Diamonsil C18(2) (250*4.6 mm, 5 μm); mobile phase: methanol; UV detector wavelength: 282 nm; detection time: 25 min.

### 2.3 RNA-Seq analysis

Total RNA of tested strains was sequenced on Illumina NovaSeq 6000 platform. Generating 150 bp paired-end raw reads. Following quality control procedures to ensure data integrity, the clean reads were obtained and aligned to the reference genome of *M. purpureus* (JGI assembly V1.0) using Hisat2 alignment tool. The mapped reads were assembled into transcripts using Stringtie. Transcripts derived from three biological replicates were analyzed using DESeq2 to identify differentially expressed genes (DEGs). Genes with an adjusted *p* value < 0.05 and fold change ≥1.5 were assigned as the DEGs. Gene ontology (GO) enrichment analysis of the DEGs was performed using the clusterProfiler package based on Wallenius noncentral hyper-geometric distribution. Kyoto encyclopedia of genes and genomes (KEGG) pathway enrichment analysis of the DEGs was performed utilizing the KOBAS database and clusterProfiler software.

### 2.4 qRT-PCR-based mRNA expression analysis

The tested mycelial tissue was collected to extract total RNA with three biological replicates. Subsequently reverse-transcribed into complementary DNA (cDNA) using a commercial kit (TOYOBO), Quantitative reverse transcription PCR (qRT-PCR) was then performed using the synthesized cDNA and SYBR Green qPCR master mix (TOYOBO), and the constitutively expressed gene *β-TUBULIN* (Protein ID: 490247) was used as an internal reference. The primers are listed in Supplementary Table 1.

### 2.5 Western blotting

To analyze histone modifications, the tested mycelial tissue were ground using liquid nitrogen, the nuclei was isolated by using the extraction buffer (20 mmol/L Tris–HCl, pH 7.5, 20 mmol/L KCl, 25% glycerol, 2 mmol/L MgCl_2_, 250 mmol/L sucrose, 0.1 mmol/L phenylmethylsulfonyl fluoride (PMSF), inhibitor) and the histone was extracted using lysis buffer (50 mmol/L Tris–HCl, pH 7.5, 150 mmol/L NaCl, 1 mmol/L EDTA, 1% Triton X-100, and 1 × protein inhibitor), experiments were conducted as previously described (Wu et al., 2024). Total histones then were separated by 15% sodium dodecyl sulfate-polyacrylamide gel electrophoresis (SDS-PAGE) gel and detected with anti-H3 (A17562, Abclonal), anti-H3Kac (A21295, Abclonal), anti-H3K4ac (A24340, Abclonal) antibodies, anti-H3K18ac (A7257, Abclonal).

### 2.6 Phylogenetic and sequence analysis

The 16 s rDNA sequence of the isolated lactic acid bacteria was subjected to blast in the National Center for Biotechnology Information (NCBI) Database. The phylogenetic analysis was performed using the aligned sequences in MEGA12 with the neighbor-joining method and 1,000 bootstrap replications.

### 2.7 Statistical analysis

All experimental data are expressed as mean ± standard deviation (SD). Statistical analyses were performed using Student’s t-test and one-way analysis of variance (ANOVA) to assess differences between mean values, with statistical significance defined as *p* < 0.05.

## 3 Results

### 3.1 Co-culture of *Lactobacillus plantarum* SC-1 and *M. purpureus* can facilitates ergosterol synthesis in *M. purpureus*

**Figure 1:**
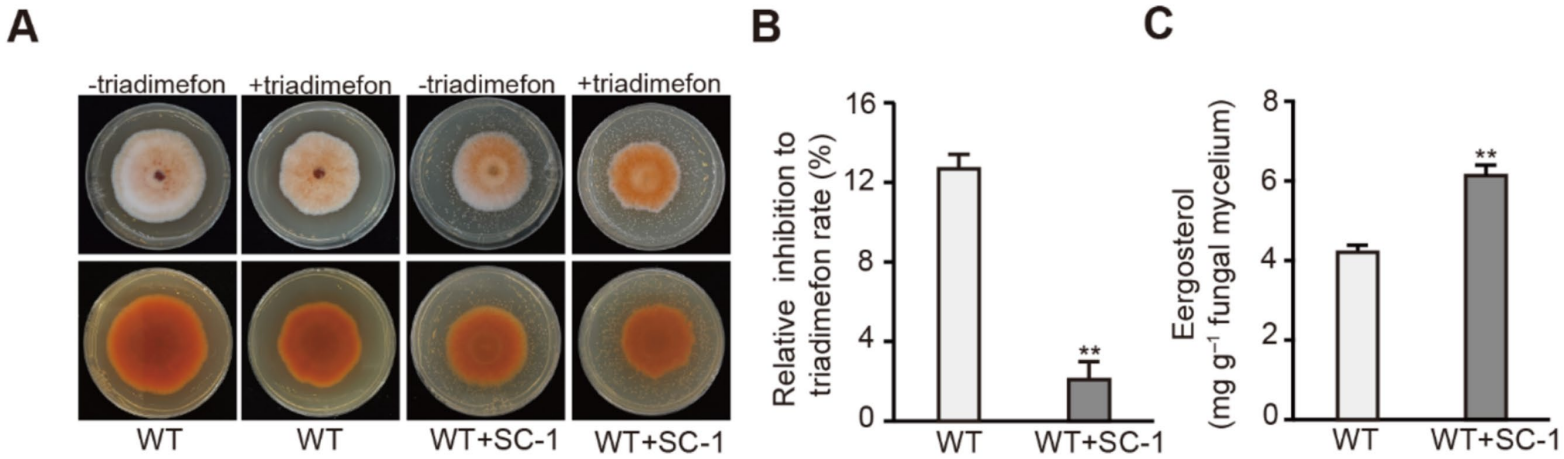
Co-culturing with SC-1 strain facilitated ergosterol synthesis in *M. purpureus*. **(A)** Colony morphology of WT and WT co-cultured with SC-1(WT + SC-1) strains grown on PDA, PDA contained triadimefon, respectively. − indicates that the medium does not contain triadimefon; + indicates that the medium contains triadimefon. **(B)** The relative inhibition to triadimefon of tested strains. The values presented represent the mean ± SD from three biological replicates. ** denotes statistically significant differences between the WT and WT + SC-1 strains at *p* < 0.01, as determined by student’s *t*-test. **(C)** The ergosterol content of tested strain grown on PDB.

To identify lactic acid bacteria that facilitate ergosterol synthesis in *M. purpureus*, we screened bacterial isolates from natural sauerkraut using triadimefon, a sterol synthesis inhibitor. we found that compared to the WT strain, WT co-cultured with the isolated SC-1 strain exhibited resistance to triadimefon, SC-1 strain can significantly inhibit the inhibitory effect of triadimefon on the growth of *M. purpureus* (Figures 1A,B). Through 16S rRNA sequencing analysis, the SC-1 strain was identified as *Lactobacillus plantarum* (*L. plantarum*), showing a high sequenece homology of 100% with known *L. plantarum* OTG002, *L. plantarum* SRCM103303 and *L. plantarum* SJ14 strains (Supplementary Figure 1). To further investigate whether *L. plantarum* SC-1 can facilitate ergosterol synthesis in *M. purpureus*, we found that compared to the WT strain, the ergosterol content of WT co-cultured with SC-1(WT + SC-1) strain increased by 45.1% (Figure 1C; Supplementary Figure 5). These results indicated that co-culture of SC-1 and *M. purpureus* strains can enhance ergosterol content in *M. purpureus*.

### 3.2 Co-culture of *L. plantarum* SC-1 and *M. purpureus* can activates ERGs in *M. purpureus*

**Figure 2:**
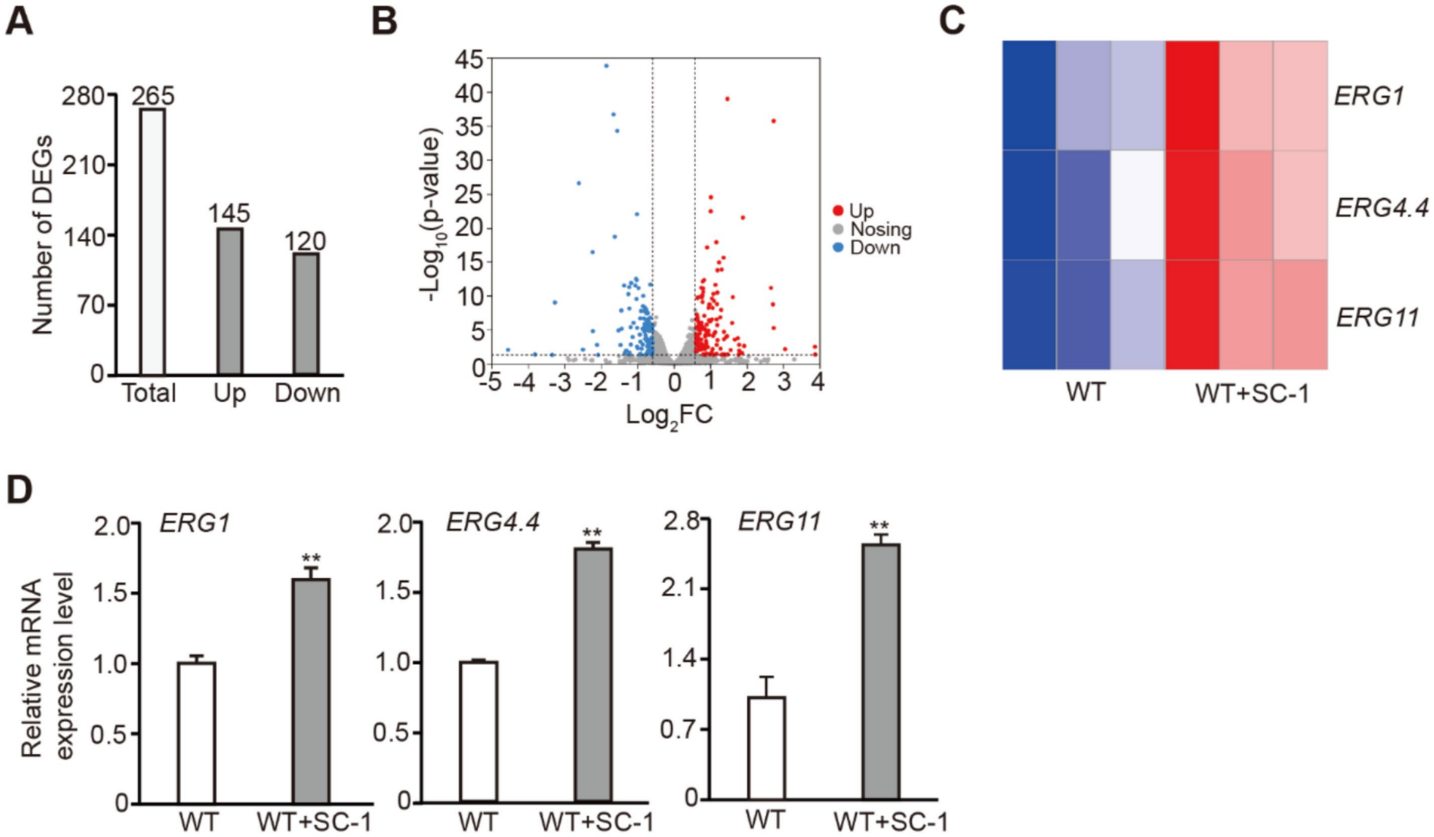
Co-cultured with SC-1 strain regulated the transcription of ERGs in *M. purpureus*. **(A)** The number of up_DEGs and down_DEGs in WT + SC-1 strain. **(B)** Volcano plot shows the expression of all DEGs in tested strain. **(C)** Heatmap shows the expressions of *ERG1*, *ERG4.4* and *ERG11* in tested strain. **(D)** Quantitative real-time polymerase chain reaction (qRT-PCR) analysis of *ERG1*, *ERG4.4* and *ERG11* expressions in tested strain. The values presented represent the mean ± SD from three biological replicates. ** denotes statistically significant differences between the WT and WT + SC-1 strains at *p* < 0.01, as determined by student’s *t*-test.

In fungi, ergosterol synthesis is usually associated with transcriptions of ERGs (Jorda and Puig, 2020; Liu J.-F. et al., 2019). To further investigate the role of SC-1 strain in facilitating ergosterol synthesis in *M. purpureus*, we analyzed the gene expression changes in WT + SC-1 strain by transcriptome sequencing. The principal components analysis (PCA) showed that WT + SC-1 strain exhibited a distinct transcriptome compared to WT strain (Supplementary Figure 2). There were 265 DEGs in WT + SC-1 strain, including 145 up-regulated DEGs (up_DEGs) and 120 down-regulated DEGs (down_DEGs) (Figures 2A,B). The transcriptome analysis of 27 ERGs in *M. purpureus* revealed that the expression levels of *ERG1*, *ERG4.4* and *ERG11* were significantly upregulated, and qRT-PCR was performed to confirm that (Figures 2C,D). Gene ontology (Go) analysis showed that up_DEGs were involved in biological processes such as transmembrane transporter activity and transporter activity (Supplementary Figure 3). Kyoto Encyclopedia of Genes and Genomes (KEGG) pathway analysis showed that up_DEGs were involved in biological processes such as thiamine metabolism and Steroid biosynthesis (Supplementary Figure 4). Taken together, these results indicated that co-culturing with SC-1 strain can facilitate ergosterol synthesis by activating the transcription of ERGs in *M. purpureus*.

### 3.3 Co-culture with *L. plantarum* SC-1 affects the establishment of histone acetylation in *M. purpureus*

**Figure 3:**
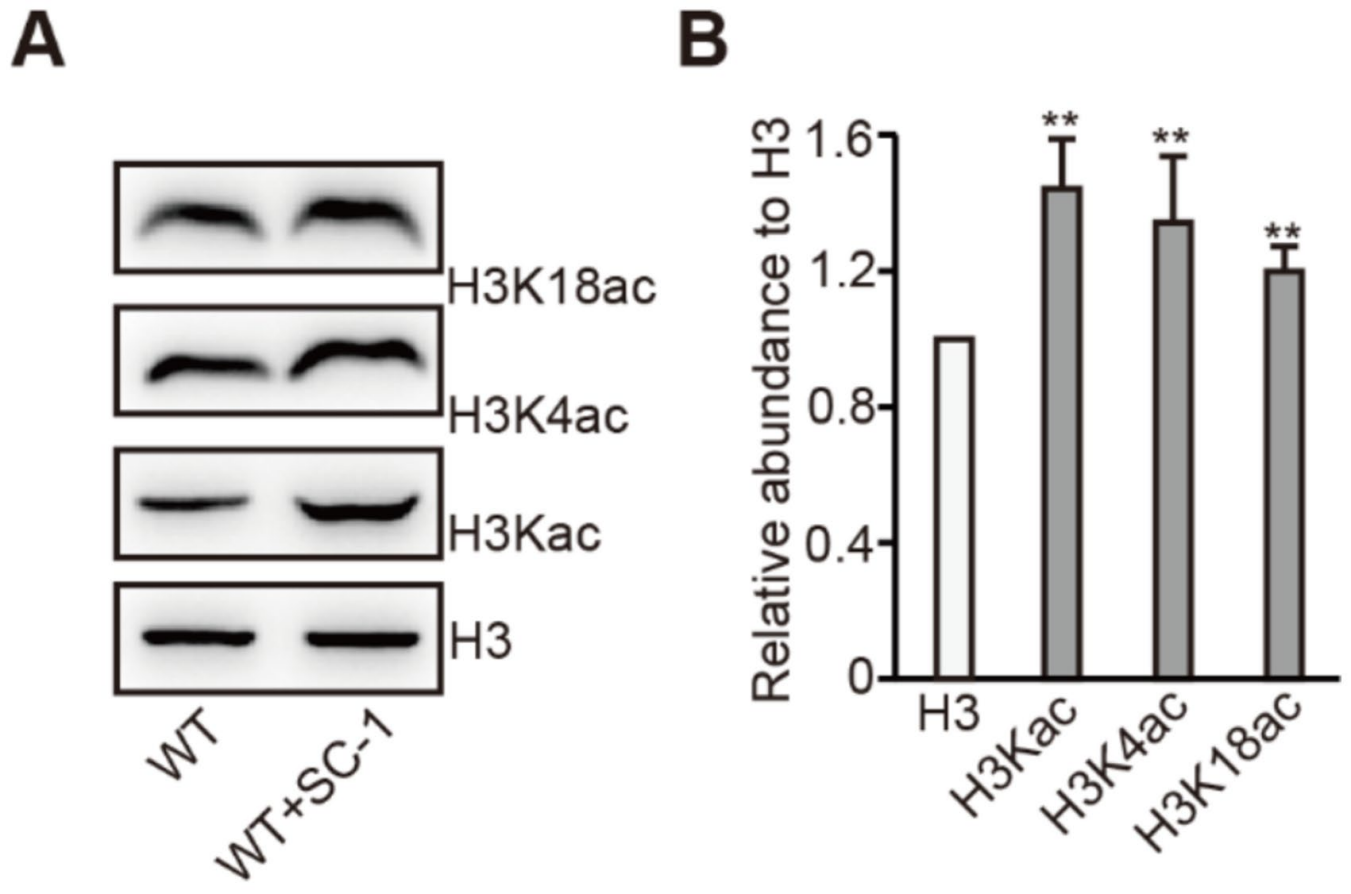
Co-cultured with SC-1 strain increases H3Kac level in *M. purpureus*. **(A)** Analysis of H3Kac, H3K4ac, and H3K18ac in tested strains by western blot analysis. **(B)** The intensities of H3Kac, H3K4ac and H3K18ac levels in tested strain. The values presented represent the mean ± SD from three biological replicates. ** denotes statistically significant differences between the WT and WT + SC-1 strains at *p* < 0.01, as determined by student’s *t*-test.

In eukaryotes, histone acetylation is typically associated with the activation of gene transcription (Graff and Tsai, 2013). To investigate whether SC-1 affects histone acetylation in *M. purpureus*, we found that compared to the WT strain, the levels of H3Kac, H3K4ac and H3K18ac in WT + SC-1 strain were significantly increased (Figures 3A,B). This result indicates that the transcriptions of ERGs were activated by the establishment of histone acetylation in *M. purpureus* treated with SC-1, thereby facilitating ergosterol synthesis.

### 3.4 MpSet1 negatively regulates ergosterol synthesis in *M. purpureus*

**Figure 4:**
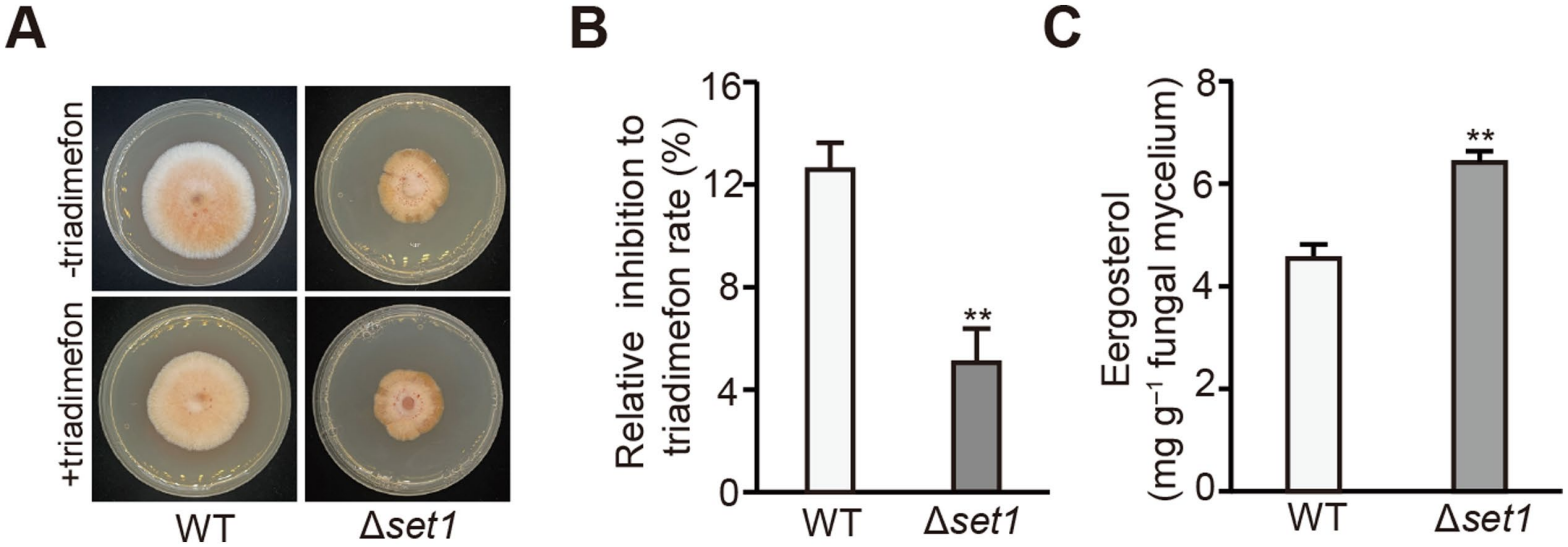
MpSet1 is crucial for ergosterol synthesis in *M. purpureus*. **(A)** Morphology of WT and Δ*set1* strains grown on PDA and PDA contained triadimefon. − indicates that the medium does not contain triadimefon; + indicates that the medium contains triadimefon. **(B)** The relative inhibition to triadimefon of WT and Δ*set1* strains grown on PDA. The values presented represent the mean ± SD from three biological replicates. ** denotes statistically significant differences between the WT and mutant strains at *p* < 0.01, as determined by student’s *t*-test. **(C)** The ergosterol content of tested strain grown on PDB.

In *Saccharomyces cerevisiae*, histone methyltransferase Set1 and histone variant Htz1 work together to negatively regulate ergosterol synthesis, however, these two proteins have functional redundancy in this process (Aslan and Özaydin, 2017). Our previous research results showed that MpSet1 can regulate MPs synthesis by catalyzing H3K4me2/3 in *M. purpureus* (Wu et al., 2024). Therefore, we hypothesized whether MpSet1 has a potential function in regulating ergosterol synthesis. To test this hypothesis, we found that compared to the WT strain, the Δ*set1* strain has a reduced sensitivity to triadimefon, and the ergosterol content was increased in Δ*set1* strain (Figures 4A–C; Supplementary Figure 6). These results show that MpSet1 can negatively regulate ergosterol synthesis in *M. purpureus*.

### 3.5 MpSet1 negatively regulates the expression of ERGs

**Figure 5:**
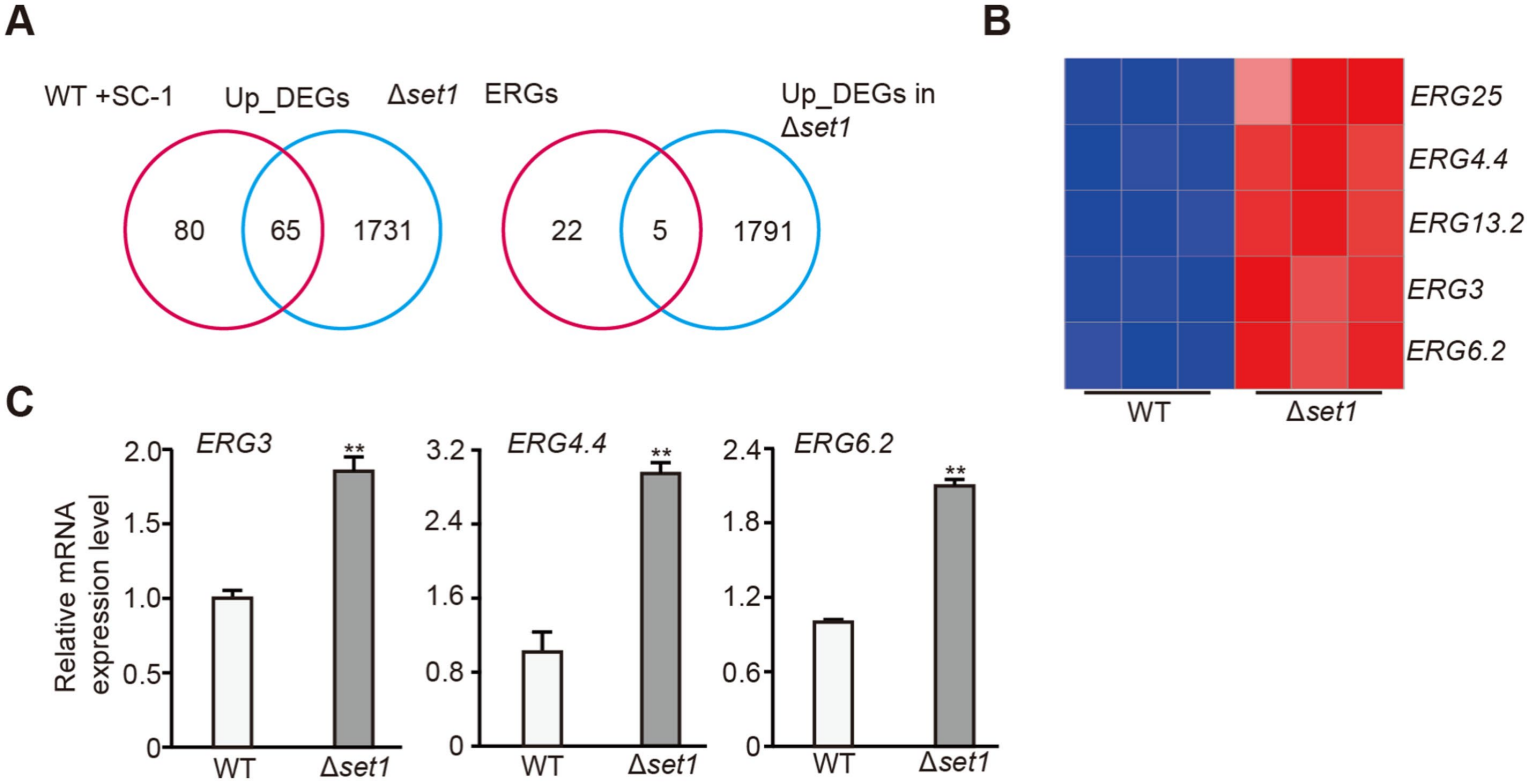
MpSet1 is crucial for the transcriptions of ERGs in *M. purpureus*. **(A)** Venn diagram shows the common up_DEGs between in Δ*set1* and WT + SC-1 strains, and the numbers of up-regulated ERGs in Δ*set1* strain. **(B)** Heatmap shows the expressions of up-regulated ERGs in WT and Δ*set1* strains. **(C)** qRT-PCR analysis of expressions of selected ERGs in WT and Δ*set1* strains. The values presented represent the mean ± SD from three biological replicates. ** denotes statistically significant differences between the WT and mutant strains at *p* < 0.01, as determined by student’s *t*-test.

In further investigate whether MpSet1 may play a role in the process of facilitating ergosterol synthesis by co-culture SC-1 and *M. purpureus*, we found that the expression levels of 65 up_DEGs among the 145 up_DEGs in WT + SC-1 strain were up-regulated in the Δ*set1* strain, and the expression levels of *ERG3*, *ERG4.4*, *ERG6.2*, *ERG13.2* and *ERG25*were significantly up-regulated in the Δ*set1* strain (Figures 5A,B). qRT-PCR detection also further confirmed that the expression levels of these ERGs were significantly up-regulated (Figure 5C). These results indicated that MpSet1 can participate in the process by which SC-1 facilitates ergosterol synthesis in *M. purpureus* by negatively regulating the expressions of ERGs.

### 3.6 MpSet1 participates in the establishment of histone H3K4ac in *M. purpureus*

**Figure 6:**
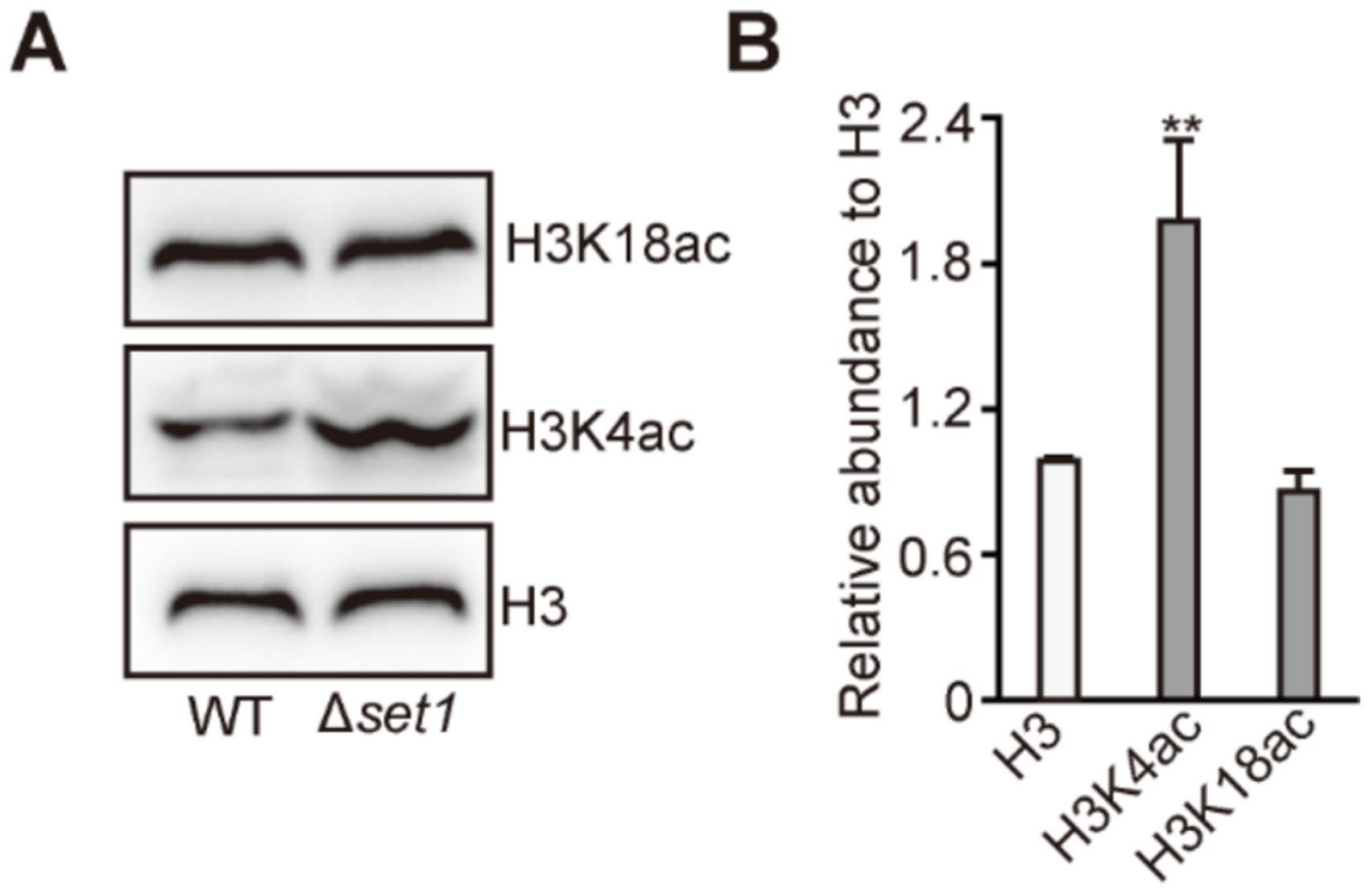
MpSet1 is important for H3K4ac establishment in *M. purpureus*. **(A)** Analysis of H3Kac, H3K4ac and H3K18ac in WT and Δ*set1* strains. **(B)** The intensities of H3Kac, H3K4ac and H3K18ac levels in tested strain. The values presented represent the mean ± SD from three biological replicates. ** denotes statistically significant differences between the WT and mutant strains at *p* < 0.01, as determined by student’s *t*-test.

In eukaryotes, Set1 typically functions as a histone H3K4 methyltransferase (Wang and Helin, 2025). To investigate whether MpSet1 affects the establishment of histone acetylation modification in *M. purpureus*, we found that compared to the WT strain, the level of H3K4ac was specifically increased in Δ*set1* strain, while the level of H3K18ac remains unchanged (Figures 6A,B). These results suggested that MpSet1 can negatively regulate the establishment of H3K4ac to regulate the expression of ERGs, and participate in the process by which SC-1 facilitates ergosterol synthesis in *M. purpureus*.

## 4 Discussion

In fungi, ergosterol, a unique steroid, plays a crucial role not only in the growth and metabolism of fungal cells but also as a precursor for various anticancer and hormone drugs (Yang et al., 2015; Rodrigues, 2018). Therefore, it is crucial to study the synthesis regulation of ergosterol and increase its production. Previous studies have shown that the synthesis pathway of ergosterol in fungi is diverse, there are multiple transcription factors in fungi, such as Upc2, Srebp and FgSr, which regulate ergosterol synthesis in fungi (Liu Z. et al., 2019; Chang et al., 2007; Vik and Rine, 2001), however, the mechanism of ergosterol synthesis in *M. purpureus* remains unclear. In this study, we performed microbial co-cultivation, RNA-seq and other methods identify a strain of *Lactobacillus plantarum* (*L. plantarum*) SC-1 isolated from sauerkraut, which could facilitate the ergosterol synthesis in *M. purpureus* (Figure 1), in addition, we found that a COMPASS core subunit Mpset1 play an important role in this process (Figures 4, 5).

In nature, microorganisms can activate the transcription of silent genes through co-culture, facilitating the synthesis of known metabolites and the production of new metabolites (Peng et al., 2021; Li et al., 2023). In industry, ergosterol production is mainly achieved through microbial fermentation (Liu J.-F. et al., 2019). In order to investigate whether ergosterol synthesis can be facilitated through microbial co-culture, our study used a co-culture system of lactic acid bacteria and *M. purpureus*, and found that WT + SC-1 strain isolated from sauerkraut can facilitate ergosterol synthesis in *M. purpureus*, which can provide a new strategy for increasing ergosterol production by microbial fermentation in industry.

To further analyzed the mechanism by which SC-1 strain facilitates ergosterol synthesis *in M. purpureus*. Transcriptome sequencing showed that the expression levels of *ERG1*, *ERG4.4* and *ERG11* were significantly upregulated in WT + SC-1 strain compared to the WT strain (Figures 2C,D). In eukaryotes, histone acetylation is usually associated with gene transcriptional activation (Kurdistani et al., 2004; Graff and Tsai, 2013). We found that compared to the WT strain, the level of H3Kac in WT + SC-1 strain was increased, and we further detected that the levels of H3K4ac and H3K18ac were increased (Figure 3), indicating that the transcriptions of ERGs were activated by affecting the establishment of histone modification in WT + SC-1 strain, thereby facilitating ergosterol synthesis. This is the first report to show that epigenetic histone modifications play a role in ergosterol synthesis. However, whether H3Kac modification can directly participate in gene transcriptional activation in this process still needs further study.

In yeast, the ergosterol synthesis is regulated by the transcription factor Upc2, which can recruit the transcriptional co-activator SAGA to the promoter of ERGs, activating the transcriptions of ERGs and facilitating ergosterol synthesis (Vik and Rine, 2001; Jorda and Puig, 2020). In addition, some regulatory factors have also been found to be able to participate in the regulation of ergosterol synthesis, such as histone methyltransferase Set1 and histone variant Htz1, which can synergistically negatively regulate ergosterol synthesis, but are functionally redundant (Aslan and Özaydin, 2017). In fungi such as *Aspergillus fumigatus*, the synthesis of sterols is similar to that in mammals and is regulated by the major transcription factor Srebp2 homologous proteins (Madhani et al., 2011). In *Fusarium graminearum*, ergosterol synthesis is regulated by the pioneer transcription factor FgSr, and Upc2 homologous protein does not participate in the process (Liu Z. et al., 2019). Our study found that MpSet1 can negatively regulate ergosterol synthesis for the first time in fungi (Figure 4). MpSet1 play an important role in the transcriptions of ERGs to regulate ergosterol synthesis (Figure 5). Loss of *MpSET1* resulted in a specific increased level of H3K4ac (Figure 6), indicating that Set1 may regulate ergosterol synthesis by negatively regulating the establishment of H3K4ac. In addition, we found that 45% up_DEGs in Δ*set1* strain were overlap with those in WT + SC-1 strain, indicating that Mpset1 plays an important role in the process of co-culturing SC-1 strain and *M. purpureus* to facilitate ergosterol synthesis in *M. purpureus*.

## 5 Conclusion

Our study performed microbial co-cultivation and revealed that co-culturing *M. purpureus* with *Lactobacillus plantarum* SC-1 isolated from sauerkraut increases ergosterol content in *M. purpureus* by 45.1%. This increase is accompanied by a differentially upregulation in the expression of *ERG1, ERG4.4, ERG11* and increased levels of H3Kac, H3K4ac, and H3K18ac. In addition, we identified that the histone methyltransferase MpSet1 in *M. purpureus* acts as a negative regulator of ergosterol synthesis for the first time, and is important for H3K4ac establishment. In addition, 45% of the up_DEGs in WT + SC-1 strain overlapped with those in Δ*set1* strain suggesting MpSet1 involved in the process by which SC-1 strain facilitates ergosterol synthesis in *M. purpureus*. Our findings provide a novel perspective on microbial fermentation strategies for enhancing ergosterol production and a new mechanism for the regulation of ergosterol synthesis in fungi.

## Data Availability

The original contributions presented in this study are publicly available. RNA‐seq datasets have been stored in the Sequence Read Archive (SRA) with the BioProject accession number: PRJNA1247207. https://www.ncbi.nlm.nih.gov/bioproject/PRJNA1247207.
