# Supplementary material for: Co-culture with Lactobacillus plantarum SC-1 facilitates ergosterol synthesis in Monascus purpureus through MpSet1-affected H3K4ac establishment
Source: Front Microbiol. 2025 Jun 23;16:1603805. doi: 10.3389/fmicb.2025.1603805 (PMC12243933; doi:10.3389/fmicb.2025.1603805)
Supplement: Supplementary file 1 [file Table_1.DOCX]

Supplementary Material

# Supplementary FIGURES and TABLES

## Supplementary FIGURES


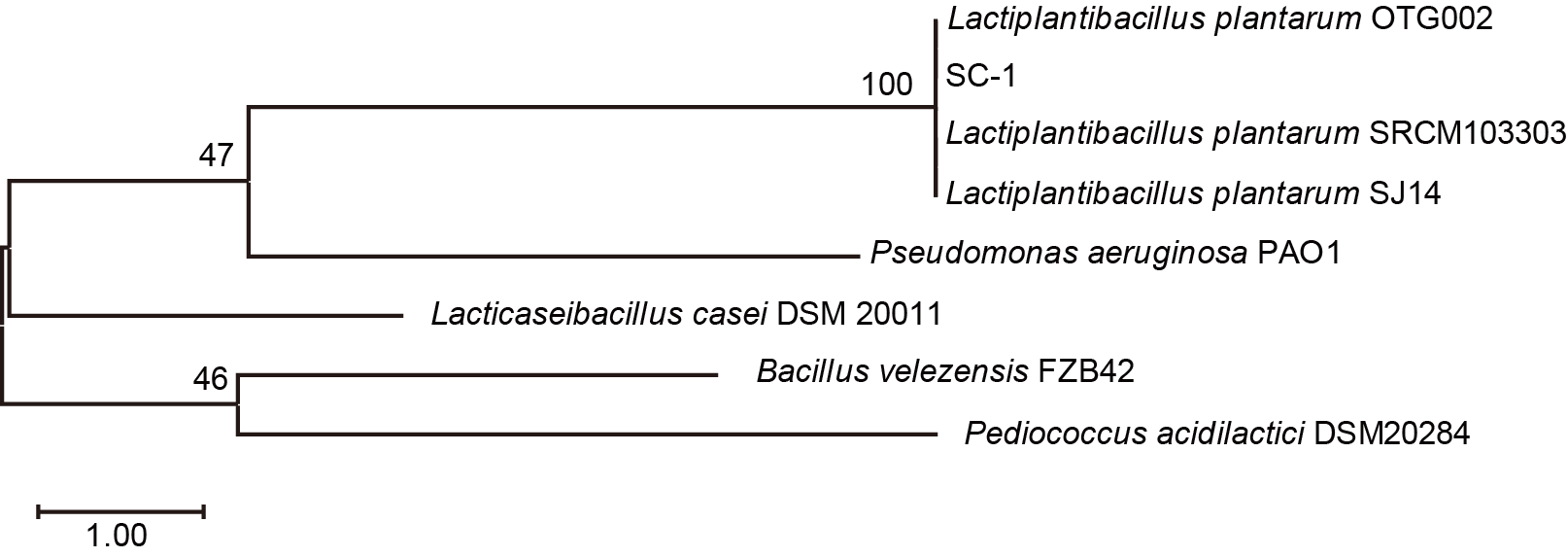


**Supplementary FIGURE 1.** Identification of *Lactobacillus plantarum* (*L. plantarum*) SC-1 strain. Phylogenetic tree generated by the neighbor-joining method with the MEGA12 software using the 16S rDNA sequences of SC-1 and following strains: *L. plantarum* OTG002, *L. plantarum* SRCM103303, *L. plantarum* SJ14, *Pseudomonas aeruginosa* PAO1, *Lacticaseibacius casei* DSM 20011, *Bacilus velezensis* FZB42 and *Pediococcus acidiactici* DSM20284.


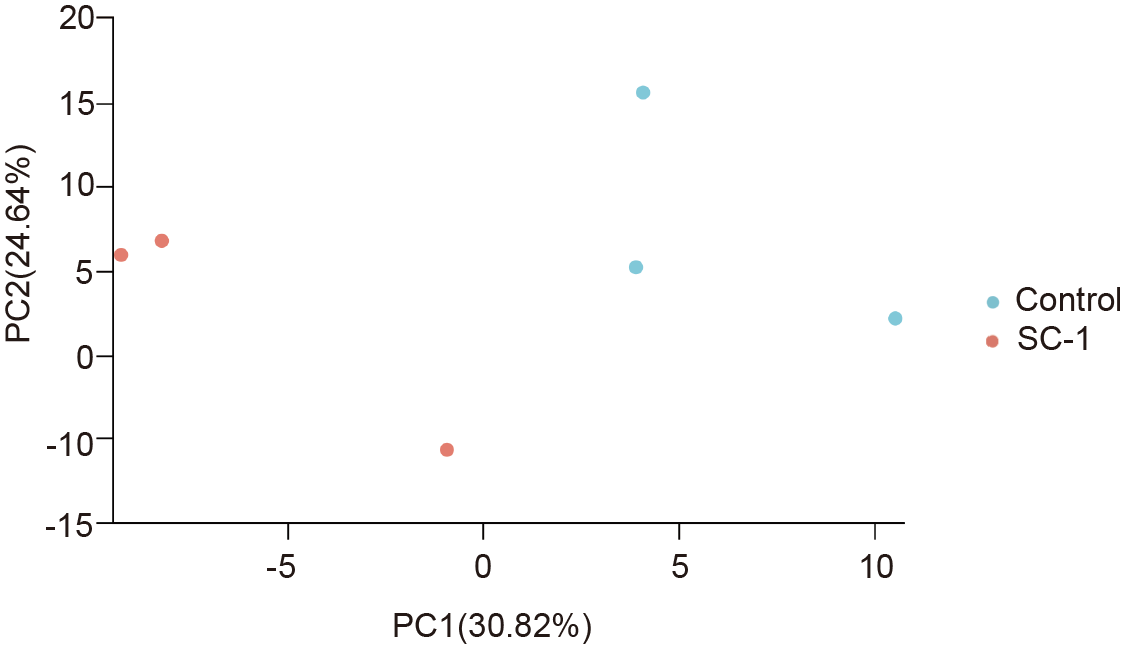


**Supplementary FIGURE 2.** Principal component analysis (PCA) of transcriptome in WT and WT co-cultured with SC-1 strain.


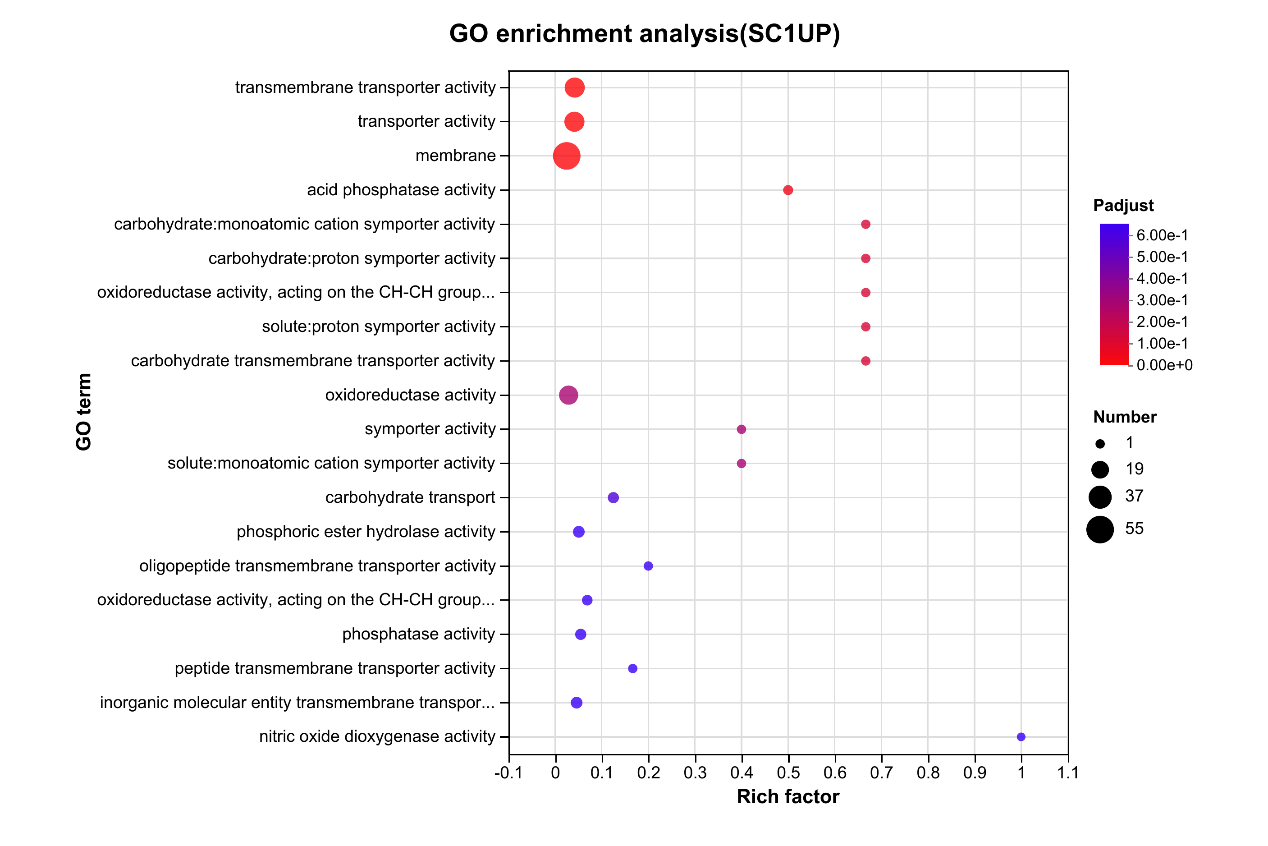


**Supplementary FIGURE 3.** GO analysis of up_DEGs in WT co-cultured with SC-1 strain compared to WT strain.


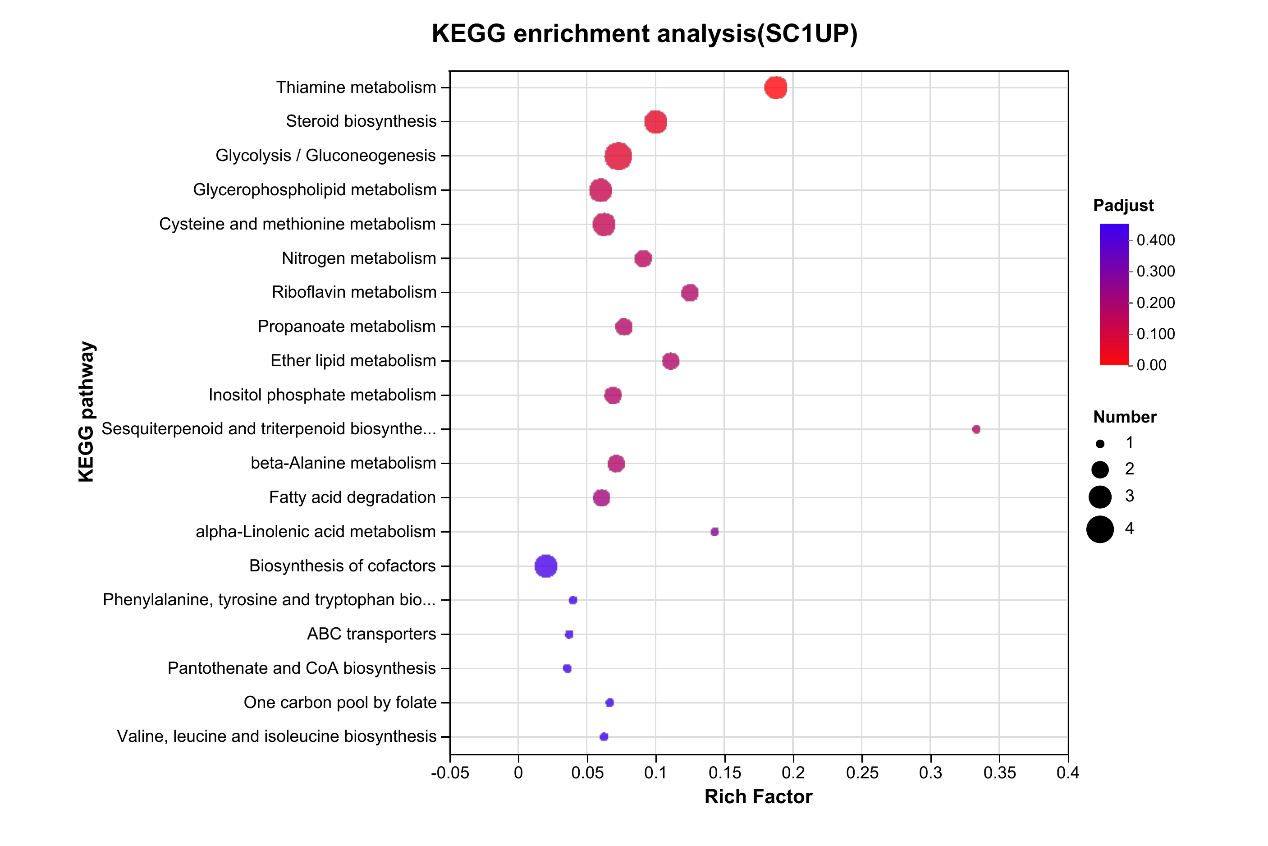


**Supplementary FIGURE 4.** KEGG analysis of up_DEGs in WT co-cultured with SC-1 strain compared to WT strain.


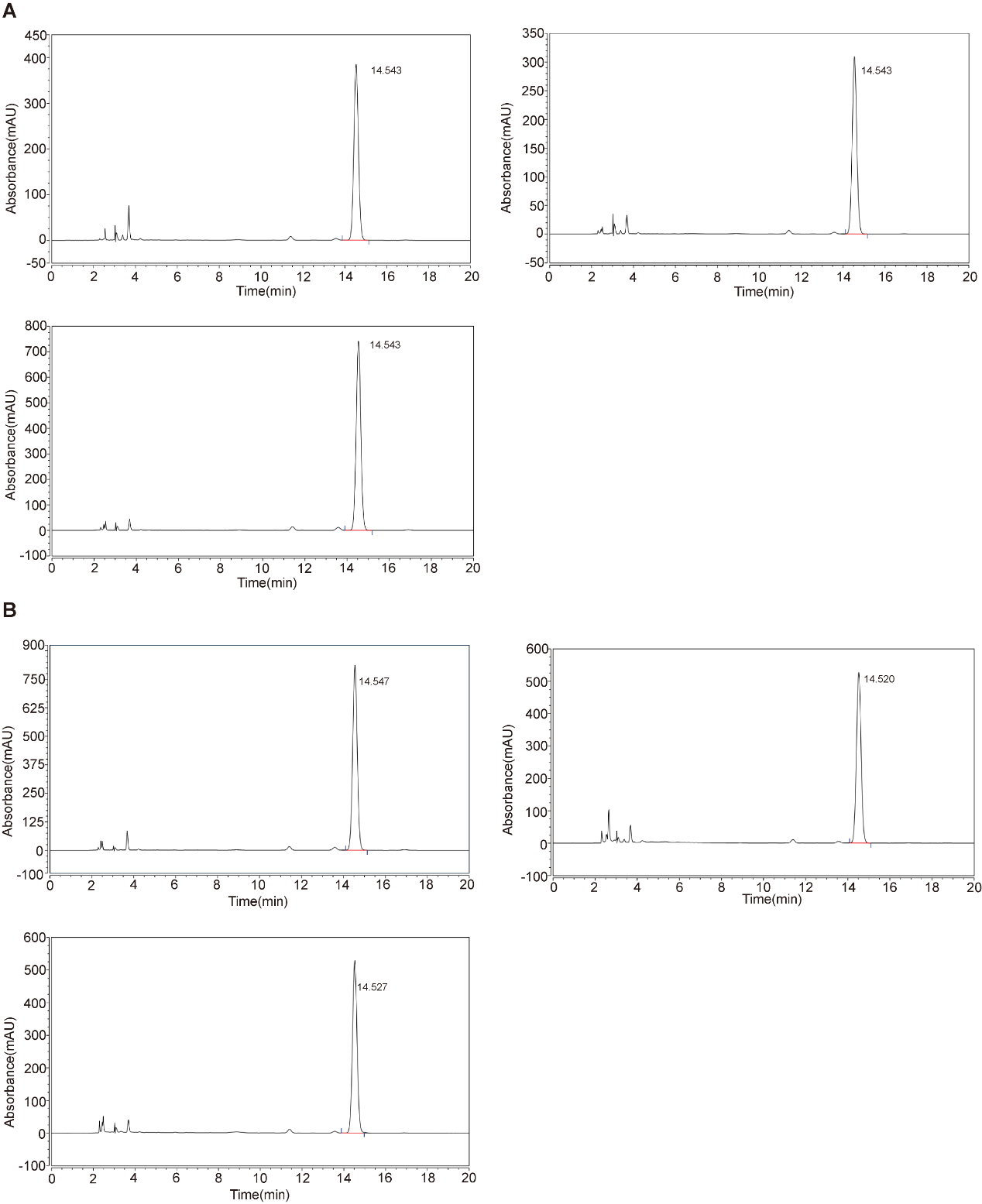


**Supplementary FIGURE 5.** Ergosterol synthesized by the WT and WT co-cultured with SC-1 strain. (a) UPLC profiles recorded at 282 nm are shown in WT strain with three biological replications. (b) UPLC profiles recorded at 282 nm are shown in WT co-cultured with SC-1 strain with three biological replications.


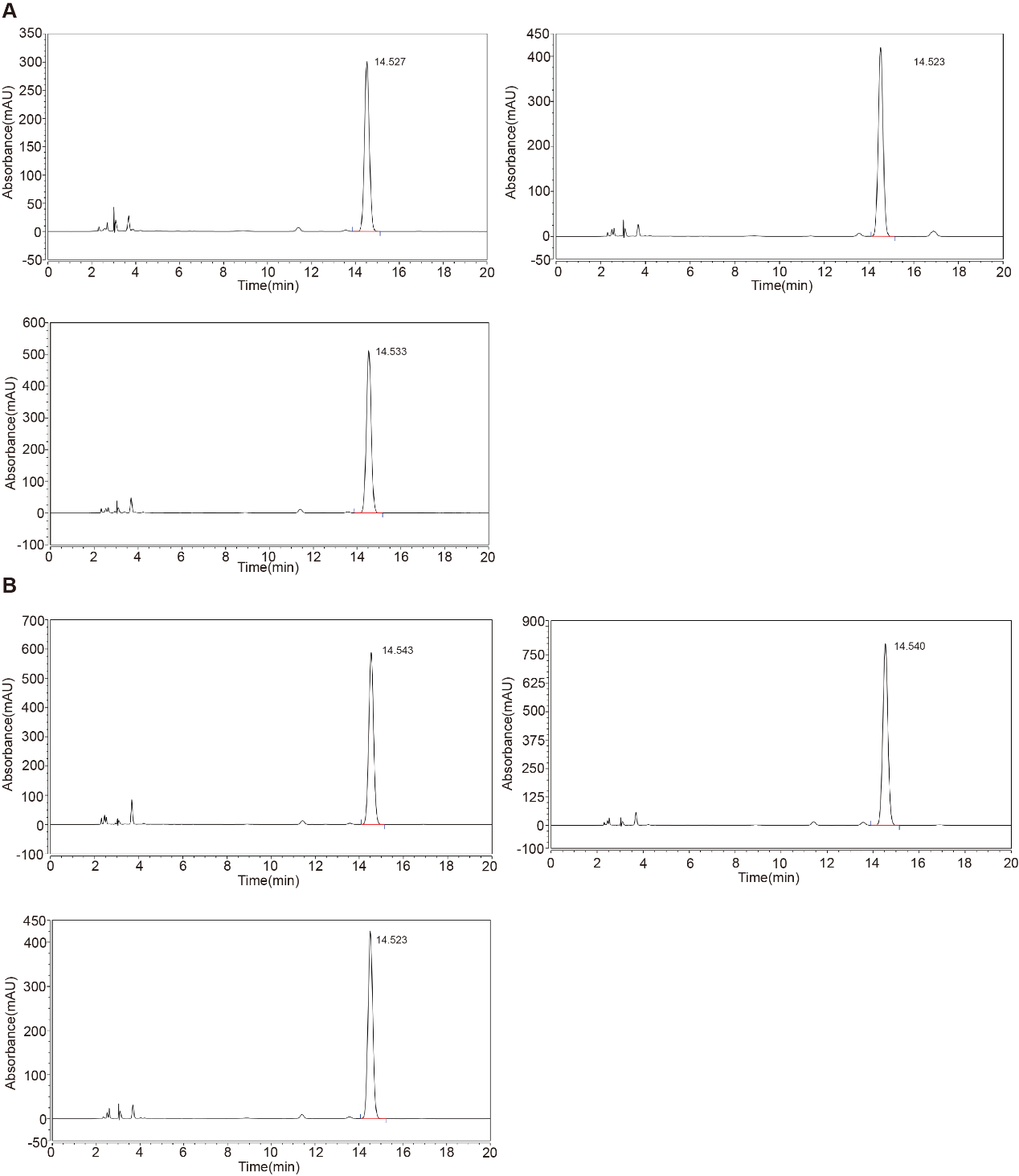


**Supplementary FIGURE 6.** Ergosterol synthesized by the WT and Δ*set1* strains. (a) UPLC profiles recorded at 282 nm are shown in WT strain with three biological replications. (b) UPLC profiles recorded at 282 nm are shown in Δ*set1* strains with three biological replications.

## Supplementary TABLE

**Supplementary TABLE 1.** Primer used in this study.

| Primer name | Sequence (5’-3’) | Use |
| --- | --- | --- |
| qMpERG1-F | TCTTTACAGTCTATTTGCTGCTGACCC | Amplifying *MpERG1* for qRT-PCR |
| qMpERG1-R | TTTCGCGGAAGAGCACCCAGA |  |
| qMpERG3-F | GTTCACCACTTTGTGGGATCGCCTA | Amplifying *MpERG3* for qRT-PCR |
| qMpERG3-R | ATTTCCTTAGTCTGTCTGTCCCACTC |  |
| qMpERG4.4-F | GATTACCAGCTTCGAGAGTCCGTTTC | Amplifying *MpERG4.4* for qRT-PCR |
| qMpERG4.4-R | CACGTATGGGATAAAGAGATAAGGAACCTG |  |
| qMpERG6.2-F | GCGCATGACGAAATTCGGGC | Amplifying *MpERG6.2* for qRT-PCR |
| qMpERG6.2-R | CCACCTCTGACAAGATGGTCCGC |  |
| qMpERG11-F | CACGAAATTGGCGAGGATGACG | Amplifying *MpERG11* for qRT-PCR |
| qMpERG11-R | GAGATTCCGCAGCTTGAAATTCTGAA |  |
| qMpβ-TUBULIN-F | CTTGCTCCGCCATCTTCCGTG | Amplifying *Mpβ-TUBULIN* for RT-PCR |
| qMpβ-TUBULIN-R | AGCTCCTGGATAGAGGTGGAGTTGC |  |
